# The need for a benchmark of the practice of cardiology

**DOI:** 10.1007/s12471-012-0253-2

**Published:** 2012-02-22

**Authors:** M. L. Simoons

**Affiliations:** Thoraxcentrum, Erasmus MC, Rotterdam, the Netherlands

**Fig. 1:**
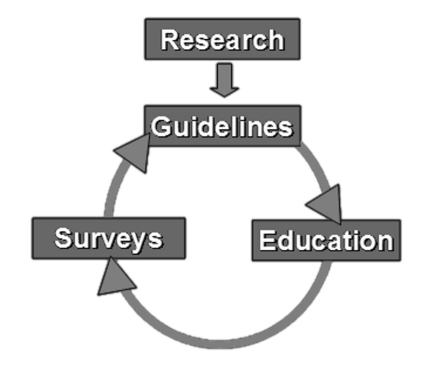
Quality development and quality assurance programmes

The Netherlands Society of Cardiology (NVvC) and the European Society of Cardiology have assumed responsibility for the quality of care in the Netherlands and in Europe. To this end, the societies organise congresses and publish journals to promote exchange of information on research and developments in cardiology. The societies develop guidelines for prevention, diagnosis and management of cardiovascular disease and they promote education programmes. Surveys and registries provide information on the actual practice of cardiology, to assess whether the guidelines are followed in clinical practice, to identify areas where improvements can be made and to verify whether the guidelines, and the clinical trials on which these are often based, really address the patients which we see in our practices (Fig. 1). Unfortunately few systematic registries of patient care are operational in our country. Therefore other sources of information should be used to gain insight into the practice of cardiology and cardiovascular medicine. A clinical trial can be such a source, since patient characteristics, treatment modalities and outcome are carefully recorded.

The report by Soedamah-Muthu and others from the Alpha-Omega trial [1] is a good example and provides information on the characteristics and the care of patients after myocardial infarction in the Netherlands. The Alpha-Omega trial investigated whether a diet with n-3 fatty acids would reduce the rate of cardiovascular events among patients after a myocardial infarction [2]. The authors should be complimented on their unique, well designed and conducted trial with different types of margarines supplemented with two different n-3 fatty acids. Although previous cohort studies indicated a protective effect of n-3 fatty acids, no such effect was found in the trial of patients who received state-of-the-art antihypertensive, antithrombotic, and lipid-modifying therapy.

**Fig. 2:**
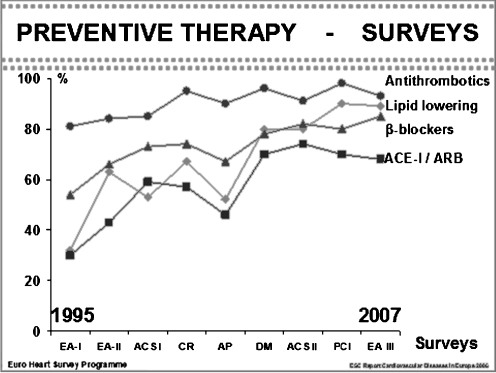
Summary of prescription of preventive therapy in different surveys by the European Society of Cardiology. EA-I, EA-II, EA-III represent EuroAspire I, II, III respectively, ACS-I and ACS-II represent surveys of Acute Coronary Syndromes, CR = survey of coronary revascularisation, AP = survey of stable angina pectoris, DM = survey of diabetes and the heart, PCI = survey of percutaneous coronary intervention. Adapted from the ESC report ‘Cardiovascular diseases in Europe 2004’ by adding data from subsequent surveys. Reproduced from Simoons: A half ounce of prevention, *Eur Heart J. 2011;32,2098–9*

The Alpha-Omega trial enrolled 4835 patients with a history of myocardial infarction from 32 hospitals between 2002 and 2006. Their mean age was 69 years and 78% were men. Overall the patients were treated intensively: in 2006, 98% received antithrombotic drugs, 87% a statin (and 3% or more other lipid-modifying drugs), 75% a beta blocker, and 59% an ACE inhibitor or angiotensin II receptor blocker. Lipid levels and blood pressure were reasonably controlled, but no information was provided on the level of control of diabetes in the 22% of patients with this disease. As in other surveys conducted in the same period (Fig. 2), the use of medication increased appropriately over the years 2002 – 2006. The authors compare these findings with the EUROASPIRE-III survey conducted in 2007 [3]. The patients in the Netherlands were older, with overall lower levels of obesity, hypercholesterolaemia, hypertension and diabetes. The patients received similar levels of antithrombotic and lipid-modifying drugs, but fewer beta blockers and ACE inhibitors were prescribed in the Netherlands. In both the EUROASPIRE survey and the Alpha-Omega trials high prevalences of smoking, obesity and diabetes were observed, which calls for action, although such lifestyle is difficult to change.

From this report different lessons can be drawn. We may be complacent, since overall cardiologists in the Netherlands who participated in the trial did treat their patients according to the guidelines in 2002–2006. The report confirms that too many patients continue their ‘bad habits’ such as smoking and too rich a diet leading to obesity and diabetes, but ‘what can we as cardiologists do about it? Habits are not easy to change’. We may question the relatively low prescription rates of beta blockers and ACE inhibitors and the NVvC and the Netherlands Institute for Continuing Cardiovascular Education (CVOI) may plan to discuss the guidelines and the underlying clinical trials again at a next congress and education programme. We may question the use of other, non-statin, lipid-modifying drugs in at least 3% of the patients since these drugs may reduce the LDL-cholesterol level, but there are no consistent data that these drugs have a favourable impact on survival or reduction of cardiovascular events. The outcomes of the ongoing IMPROVE-IT trial to assess the value of ezetimibe in secondary prevention are eagerly awaited. Indeed, we might sit back and be reassured that in our practice we need just a bit more attention to further improve our secondary prevention measures.

However, to my regret, no data on the 32 individual practices are presented in the report. To assess the quality of our practices we cannot hide behind overall data from our country, even if one third of the hospitals in the Netherlands have provided these data. We need to compare the actual data from our own practice, from our own hospital, with others to identify areas for our own improvement. We need actual data from 2011 and 2012 and should not be satisfied with a review of data collected several years ago because practices change, as illustrated again in Fig. 2 and in the report by Soedamah-Muthu and others [1]. I do believe that most cardiologists in the Netherlands provide a good service, but I have no access to data on individual practices to verify this belief. Together we fail to collect and provide such data on patient care, on the use of diagnostic resources and on specific therapeutic procedures in interventional cardiology and clinical electrophysiology. What are the outcomes of these procedures in different hospitals? Which complications do occur and how frequently? Insight into such data, in actual data from 2011 and 2012, is required to further improve the quality of our care.

A registry of all procedures, outcome and complications is a requirement for licences for interventional cardiology and other procedures. The National Cardiovascular Data Registry (NCDR) has been created for this purpose. Registries for pacemaker and ICD implantation, for percutaneous coronary interventions and for percutaneous aortic valve implantation have been developed, but I understand that data collection is slow, that existing registries in different hospitals are not fully consistent and that no agreement has been reached on the type of reports which professionals and the public may expect. Yet, agreement on the content of many registries was reached at the European level several years ago [4] and a first report was published from the hospitals participating in the Euro-Heart Survey / Zorgprogramma Nederlandse Hartstichting [5]. I look forward to similar reports with up-to-date information from all hospitals in the Netherlands. Some other countries have been more successful in this matter, for example Sweden with the ongoing RIKS-HIA registries which can be found on the internet with many details on the performance of all Swedish hospitals [6]. The NVvC should ensure that together we will collect and provide the information which is required to benchmark our practices. We should overcome the hurdles and ‘cold feet’ which have blocked progress in this field. The report from the Alpha-Omega trial confirms that we do reasonably well, but we can do even better if we all participate in a proper benchmarking system.

Prof. dr. Maarten L. Simoons

Thoraxcentrum, Erasmus MC, Rotterdam, the Netherlands
